# Peroxisome Proliferator-Activated Receptor α Activation Is Not the Main Contributor to Teratogenesis Elicited by Polar Compounds from Oxidized Frying Oil

**DOI:** 10.3390/ijms18030510

**Published:** 2017-02-27

**Authors:** Yu-Shun Lin, Ting-Yi Lin, Jia-Jiuan Wu, Hsien-Tsung Yao, Sunny Li-Yun Chang, Pei-Min Chao

**Affiliations:** 1 Department of Nutrition, China Medical University, Taichung 404, Taiwan; chriscats211@yahoo.com.tw (Y.-S.L.); tingyilin1025@gmail.com (T.-Y.L.); jjwu@mail.cmu.edu.tw (J.-J.W.); htyao@mail.cmu.edu.tw (H.-T.Y.); 2 Graduate Institute of Biomedical Sciences, China Medical University, Taichung 404, Taiwan; liyunchang@mail.cmu.edu.tw

**Keywords:** PPARα, polar compounds, oxidized frying oil, retinoic acid, teratogenesis

## Abstract

We previously reported that polar compounds (PO) in cooking oil are teratogenic and perturbed retinoic acid (RA) metabolism. Considering PO as a potent peroxisome proliferator-activated receptor α (PPARα) activator, this study aimed to investigate the role of PPARα in PO-induced teratogenesis and disturbance of RA metabolism. Female PPARα knockout or wild type mice were mated with males of the same genotype. Pregnant mice were fed a diet containing 10% fat from either fresh oil (FO) or PO from gestational day1 to day18, and killed at day18. The PO diet significantly increased the incidence of teratogenesis and fetal RA concentrations, regardless of genotype. Though PPARα deficiency disturbed maternal RA homeostasis, itself did not contribute to teratogenesis as long as FO diet was given. The mRNA profile of genes involved in RA metabolism was differentially affected by diet or genotype in mothers and fetuses. Based on hepatic mRNA levels of genes involved in xenobiotic metabolism, we inferred that PO not only activated PPARα, but also altered transactivity of other xenobiotic receptors. We concluded that PO-induced fetal anomalies and RA accumulation were independent of PPARα activation.

## 1. Introduction

Many environmental contaminants and chemicals (including heavy metals, polychlorinated biphenyls, radiation, and illicit drugs) are known to cause developmental toxicity, with warnings given to women of childbearing age [1,2]. However, we know little regarding potential risks associated with foods. Oxidized frying oil (OFO) is essentially an environmental pollutant that can be regarded as a xenobiotic since it is foreign to the body, lipophilic and induces expression of detoxifying CYP450 monoxygenase and phase II conjugation enzymes to facilitate its own catabolism [3]. We recently reported that OFO, specifically, the polar fraction or polar compounds (PO), is teratogenic, and that this effect was associated with disturbed retinoic acid (RA) metabolism in pregnant mice and their fetuses [4]. Nevertheless, underlying mechanisms of disturbed RA metabolic gene expression by PO remain unknown.

It is noteworthy that RA is a well-characterized morphogen; by binding to the RA receptor (RAR) and retinoid X receptor (RXR), it controls a network of genes (e.g., homeobox gene *HoxB1*) that are critical for morphogenesis [5]. During embryonic development, delicate spatio-temporal regulation of RA concentrations is required. Endogenous RA is synthesized from retinol in two steps. The first step involves oxidation of retinol into retinal. Many enzymes, including those in the alcohol dehydrogenase and short-chain dehydrogenase/reductase families, have this activity [6], with retinol dehydrogenase (RDH) 10 regarded as the most important enzyme during embryonic development [7]. However, this reaction can be reversed by enzymes with retinal reductase activity. One of which, dehydrogenase/reductase SDR family member 4 (DHRS4), is a peroxisomal enzyme putatively regulated by peroxisome proliferators (PPs) [8]. The opposing activities of RDH and retinal reductase appear to control the steady-state levels of retinal, the immediate precursor for RA biosynthesis [9]. The second step in RA synthesis involves irreversible conversion of retinal into RA by retinal dehydrogenase (RALDH). Of the various isoforms, RALDH1, 2 and 3 (also known as ALDH1A1, 1A2 and 1A3, respectively) were reported (based on gene knockout studies) to be essential for embryonic development [10]. Although many CYP450 enzymes exhibit RA catabolic activity (e.g., CYP1, CYP2 and CYP3) [11], CYP26A1, CYP26B1, CYP26C1, and CYP2C39 are the most highly expressed isoforms in the mouse liver which produce 4-oxo-, 4-OH– or 18-OH-RA for excretion [12,13,14].

When foods are deep fried, a series of reactions including hydrolysis, thermal oxidation, polymerization, cyclization and fission, occur in the frying oil [15], which is ingested along with foods. Fresh cooking oil is relatively non-polar, as it consists of non-oxidized triglyceride. However, polarity increases with extended frying, due to accumulation of hydrolytic products and of altered triglyceride with at least one oxygenated functional group (e.g., epoxides, ketones or alcohols), on esterified fatty acids [16]. Therefore, PO (i.e., oxidized components) is used globally as an index for quantification of altered components in used oils. For food safety concerns, most countries adopt the limitation that PO in the used frying oils for human consumption should not be higher than 25% of the oil weight [16].

We previously reported that PO is a major contributor to peroxisome proliferator-activated receptor α(PPARα) activation by OFO [4]. As a nuclear receptor, PPARα has a pivotal role in regulating systemic lipid homeostasis by modulating an array of target genes participating in lipid catabolism and lipoprotein assembly and transport [17]. Some genes involved in RA metabolism (e.g., *Dhrs4*, *Cyp2C*, and *Cyp26*) were reported to be affected by PPARα activators [8,18,19]. Furthermore, exposure to xenobiotics di(2-ethylhexyl)phthalate or to perfluorooctanoic acid activates PPARα, causing developmental toxicity or teratogenesis, with PPARα regarded as being required for these defects [20,21,22]. Conversely, teratogenesis was absent when other PPARα activators, such as fibrate-class hypolipidemic drugs, were given to pregnant mice [23], Therefore, the objective of the present study was to investigate the role of PPARα in PO-mediated teratogenesis and RA disturbance using PPARα knockout (KO) mice.

## 2. Results

### 2.1. Effects of PO Diet or PPARα Deficiency on Embryotocixity and Reproductive Characteristics

The oil quality index of PO used had an acid value and conjugated diene that were 52- and 12-fold, respectively those of the FO (Table 1). When WT or KO females were fed a diet containing 10% fat from fresh soybean oil (FO) or PO from day1 to day18 of pregnancy, feed intake was equivalent among groups (5.02 ± 0.14 g/day), although body weight (including uterine and fetus weight) was significantly and independently affected by genotype (*p* < 0.05), but not by diet. In accordance with early-onset obesity is characterized in PPARα KO females [24], mice in groups FO(KO) and PO(KO) consistently had a greater body weight than the wild type during pregnancy (Figure S1). However, for maternal body weight gain (excluding uterine and fetus weights), values were significantly lower, regardless of genotype, for the PO diet compared to the FO diet (Table 2). Liver enlargement is one of the pleiotropic responses of PPARα activation [25]. There was a significant interaction between diet and genotype for maternal liver weight/body weight percentage at day18, i.e., the PO diet significantly increased relative liver weight in WT, but not in KO mice.

Regarding variables associated with embryotoxic and reproductive effects (Table 2), neither diet nor genotype affected numbers of corpora lutea, implantations, total fetuses, live or dead fetuses, pre- or post-implantation loss per litter, or litters with three or more dead fetuses or resorption of the entire litter. Body weight, liver weight, and mortality rate of fetuses were unaffected by diet or genotype, although placenta weight was significantly increased by the PO diet in both WT and KO mice (Table 3).

### 2.2. Effects of PO Diet or PPARα Deficiency on Teratogenesis

In contrast to no externally apparent congenital anomalies for FO(WT) and FO(KO) mice, scattered cases of eye defect, edema, brain defect (anencephaly or microcephaly), haematoma, surface shriveling, spina bifida, and limb defects (without hind limbs) were detected in fetuses from both genotypes if their mothers received the PO diet (Table 3). The external congenital abnormalities occurred in this study were shown in Figure S2. Regardless of the genotype, the PO diet significantly increased the incidences of superficial haematoma, shrivelling and total abnormalities, i.e., the number of fetuses with at least one abnormality. There was no interaction between both factors.

### 2.3. Effects of PO Diet or PPARα Deficiency on Vitamin A Status

**Figure 1:**
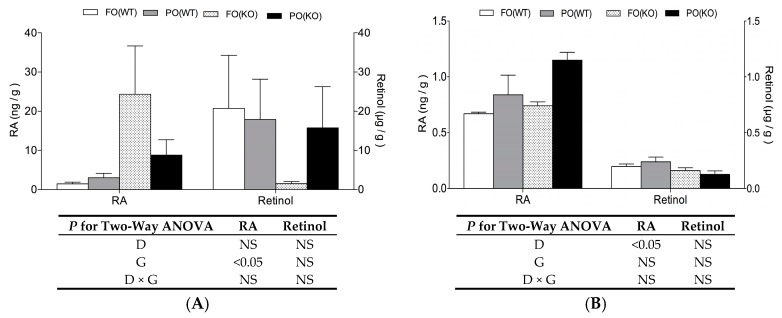
Retinol and retinoic acid (RA) concentrations in maternal liver (**A**) and fetus (**B**) of WT and KO mice receiving FO or PO diets during pregnancy. Samples were collected at pregnancy day18. Data are mean ± SEM, *n* = 5. Results of two-way ANOVA are shown in table (D, diet; G, genotype; D × G, interaction; NS, not significant).

Concentrations of retinol (including retinol and retinyl ester) and RA were measured in maternal livers or their fetuses recovered on day18 (Figure 1). In maternal liver, PPARα deficiency caused RA to significantly increase. In that regard, there was a 17- and 3-fold RA accumulation (with corresponding decreases in retinol concentrations), in the KO compared to the WT mice when they were subjected to the FO or PO diet, respectively (Figure 1A). However, for whole-fetal quantification, PPARα deficiency had no effect on RA or retinol concentration (Figure 1B). For whole-fetal RA concentration, it was the PO diet, rather than genotype, that significantly and independently increased RA concentrations.

### 2.4. Effects of PO Diet or PPARα Deficiency on RA Metabolic Gene Expression

**Figure 2:**
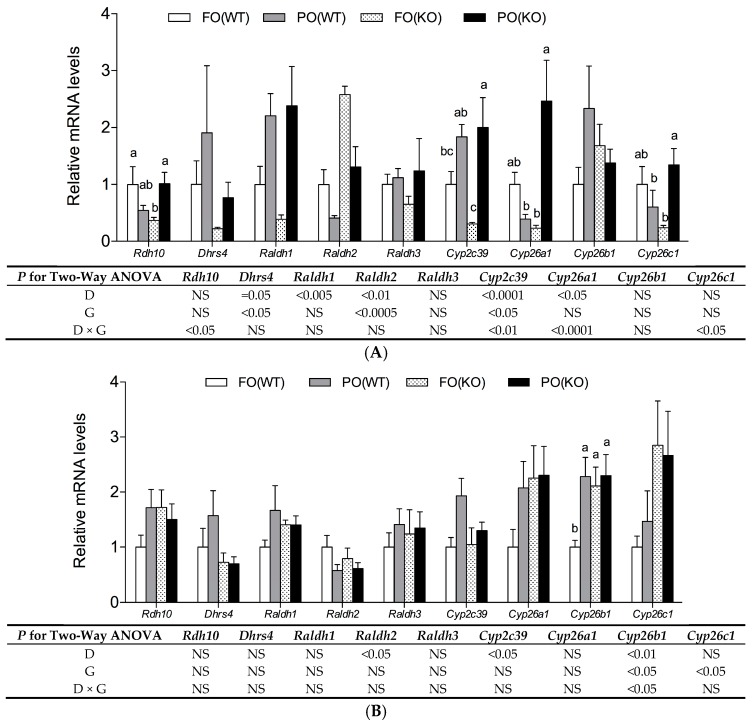
Levels of mRNA for genes associated with RA metabolism in maternal liver (**A**) and fetal liver (**B**) of WT and KO mice receiving FO or PO diets during pregnancy. Samples were collected at pregnancy day18. The value for the FO(WT) group was taken as 1. Data are mean ± SEM, *n* = 5. Results of two-way ANOVA are shown in table (D, diet; G, genotype; D × G, interaction; NS, not significant). When there was a significant interaction between D and G, the significance of differences among groups was further analyzed by one-way ANOVA and Duncan’s multiple range test; ^a–c^ Values without a common superscript letter differed (*p* < 0.05).

Hepatic mRNA levels of genes participating in RA synthesis and catabolism of mothers and their fetuses at day18 are shown (Figure 2). In maternal liver, a PO diet significantly and independently increased mRNA levels of *Dhrs4*, *Raldh1* but reduced mRNA levels of *Raldh2* (Figure 2A). The mRNA levels of *Dhrs4* and *Raldh2* were also significantly and independently reduced and increased, respectively, by PPARα deficiency. In WT mothers, a PO diet decreased *Rdh10*, *Cyp26a1* and *Cyp26c1* slightly, increased *Cyp2c39* slightly on mRNA levels. However, in KO mothers, PO diet increased *Rdh10*, *Cyp2c39*, *Cyp26a1* and *Cyp26c1* significantly on mRNA levels. Therefore, there were significant interactions of diet and genotype for these transcripts.

In fetal livers, a PO diet significantly and independently reduced levels of *Raldh2* mRNA, but increased those of the *Cyp2c39* (Figure 2B). PPARα deficiency increased *Cyp26c1* mRNA levels. Furthermore, a significantly higher *Cyp26b1* transcripts was observed in fetuses of PO(WT), FO(KO) and PO(KO) groups compared to the FO(WT) group (*p*-interaction < 0.05).

### 2.5. Effects of PO Diet or PPARα Deficiency on Transactivity of Xenobiotic Receptors

**Figure 3:**
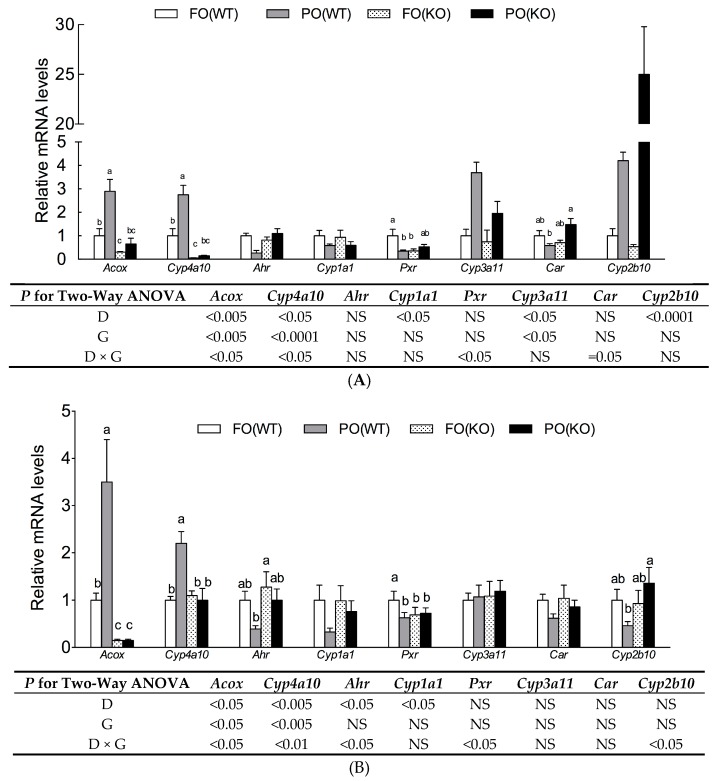
Levels of mRNA for genes associated with xenobiotic receptor activity in maternal liver (**A**) and fetal liver (**B**) of WT and KO mice receiving FO or PO diets during pregnancy. Samples were collected at pregnancy day18. The value for the FO(WT) group was taken as 1. Data are mean ± SEM, *n* = 5. Results of two-way ANOVA are shown in table (D, diet; G, genotype; D × G, interaction; NS, not significant). When there was a significant interaction between D and G, the significance of differences among groups was further analyzed by one-way ANOVA and Duncan’s multiple range test; ^a–c^ Values without a common superscript letter differed (*p* < 0.05).

It has been established that PPARα, aryl hydrocarbon receptor (AhR), constitutive androstane receptor (CAR), and pregnane-X receptor (PXR) are transactivated by xenobiotics. For PPARα activity, the mRNA levels of *Acox* and *Cyp4a10*, two typical PPARα target genes were measured in livers of mothers (Figure 3A) and fetuses (Figure 3B) at day18. These two transcripts were upregulated by the PO diet, but downregulated by PPARα deletion. The PO upregulated *Acox* and *Cyp4a10* gene expression were only significant in the WT, but not in KO mice, thus leading to a significant interaction of diet and genotype.

Levels of mRNA for *Ahr*, *Pxr* and *Car*, and their target genes, *Cyp1a1*, *Cyp3a11* and *Cyp2b10*, respectively, were also measured. In maternal and fetal livers, a PO diet suppressed *Cyp1a1* regardless of genotype, in line with a suppression of AhR transactivation by PO as previously demonstrated in a reporter assay [4]. Though the mRNA levels of *Pxr* in FO (WT) dams and fetuses were the highest compared to their counterparts in other groups (*p*-interaction < 0.05), *Cyp3a11* was upregulated by PO diet in dams. For CAR transactivity, PO-induced *Cyp2b10* expression were only present in maternal, but not in fetal liver.

## 3. Discussion

In accordance with our previous study, ingestion of PO in pregnant WT mice had no significant effect on early embryonic implantation, although it induced congenital malformations in offspring [4]. In addition, PO altered mRNA profiles of genes involved in RA metabolism. In this study, KO mice lacked typical PO-induced PP responses, e.g., hepatomegaly and upregulation of *Acox* and *Cyp4a10* expression, consistent with a lack of functional PPARα. Although PO has been reported to be a potent PPARα activator and administration of PO to pregnant females activates PPARα signaling in both mothers and fetuses (Figure 3), the present study provided clear evidence that the pathogenesis of PO-induced teratogenesis and fetal RA excess outcome did not involve PPARα activation.

Links between environmental pollutants causing teratogenicity and altered retinoid physiology have been well recognized [26,27], though the mechanisms underlying disruption of retinoid signaling by pollutants have not been elucidated. We were apparently the first to propose that PO-mediated malformations were associated with perturbed RA metabolism in mothers and fetuses. Indeed, when pregnant mice were given PO throughout gestation, gene expression pattern for RA synthesis (e.g., *Rdh10*, *Dhrs4*, *Raldh1*, and *Raldh2*) as well as RA catabolism (e.g., *Cyp2c39*, *Cyp26a1*, *Cyp26b1* and *Cyp26c1*) in mothers and fetuses deviated from that of their peers (Figure 2 and [4]). Likewise, that the same enzyme families (alcohol dehydrogenase, short-chain dehydrogenase/reductase and RALDHs) were shared for alcohol metabolism and RA synthesis, alcohol might be a competitor for RA synthesis. In that regard, fetal alcohol spectrum disorder, which includes multiple teratogenic effects in human embryos exposed to alcohol, is ascribed to suppressed activity of RALDH2 [28], the predominant enzyme responsible for generating RA in early embryos [29]. Reduced RA concentrations and impaired RA signaling due to an inhibited RALDH2 were regarded as causing alcohol-induced embryonic malformations [28].

One unexpected finding of the present study is PPARα deficiency, by itself, caused substantial increases in hepatic RA concentrations in pregnant mice, irrespective of diet (Figure 1A). Perhaps this RA build-up in maternal liver of KO mice was due to downregulated *Dhrs4* and upregulated *Raldh2* expression induced by PPARα deficiency, thus providing more substrate (retinal) and a higher capacity for RA synthesis. However, this genotype effect on *Dhrs4* and *Raldh2*, as well as RA excess, was absent in fetuses. We inferred that there were distinct regulatory mechanisms for RA metabolism in mothers versus their developing embryos. This was not unexpected, since spatial and temporal regulation of RA metabolic enzymes is highly specific and dynamic in embryos to insure RA concentration is tightly controlled [14].

Although RA accumulation was seen in liver of PPARα deficient mothers, it did not contribute to fetal RA surplus, nor did it cause teratogenesis, since no malformations were detected in fetuses of group FO(KO), which had an equivalent RA concentration as the normal control, i.e., fetuses of group FO(WT). It is well known that exogenous RA can cross the blood-placenta barrier and affect developing embryos [30]; therefore, we suspected that increased *Cyp26b1* and *Cyp26c1* mRNA levels in liver of the KO fetuses might be part of the defensive responses to excess RA from their mothers. To our knowledge, vitamin A metabolism in PPARα-null mice has not been reported, perhaps due to the lack of teratogenic phenotype.

It is believed that the precursor for RA synthesis (i.e., retinol) is provided by maternal circulation, and fetus synthesizes its own RA, which is tightly regulated in a spatio-temporal manner, by expressing synthetic/catabolic enzymes. Increased availability of locally synthesized RA would generate a RA diffusion gradient, working in concert with the RAR/RXR nuclear receptors, that is required for differentiation and development of organs surrounding sites of synthesis [31,32]. During embryonic development, RA requirements were markedly increased at embryonic day7.5–day8.5, when *Raldh2* mRNA and RA were first detectable in embryonic mesoderm [29]. Currently, we are unable to explain PO-induced fetal RA accumulation outcome based on the hepatic mRNA profile measured only at the endpoint. This drawback limits the interpretation since the significant defects observed would have resulted from disruptions in much earlier developmental events and we have missed some critical time points by analyzing only day18. In future studies, transgenic mice with an RA response element (RARE)-driven reporter combined with in situ hybridization for detecting critical enzymes involved in RA metabolism would be helpful in addressing this issue.

Among three enzymes that synthesize RA, RALDH2 is more broadly expressed in embryos and has frequently been used to represent RA signaling pattern across embryogenesis [29]. Feedback regulation of *Raldh2* by RA has been demonstrated, since a RARE located in *Raldh2* promoter was directly repressed by RA [33]. In addition, CYP26 protected embryos from excess RA exposure [13]. Compared to *Cyp26b1* and *Cyp26c1*, *Cyp26a1* is highly inducible by RA via the synergistic responses of two RAREs within its promoter region [34]. Therefore, we considered that the down-regulated *Raldh2* and up-regulated *Cyp26* genes by PO might be a defensive response to embryonic RA excess. We previously supplemented RA in the meternal PO diet, i.e., 100 µg/g at day7.5–day8.5 and shift to 250 µg/g at day8.5–day18 as being suggested in ref [7]. However, it failed to rescue and even worsened the teratogenesis outcome (with 72% incidence of externally visible abnormalities accompanied with an increased resorption rate). To confirm the causal effect of PO-induced embryonic RA accumulation on teratogenesis, putting PO-exposed dams on a vitamin A-deficient diet to see if the teratogenesis could be rescued is required.

The PPARα was not the only target affected by PO. We had demonstrated that PO inhibited AhR in a transactivation assay and in the liver of pregnant mothers and their fetuses [4]. In the present study, there was evidence that PO might increase transcriptional activity of CAR and PXR in maternal liver, although this effect in fetuses was unclear (Figure 3). Furthermore, 2,3,7,8-Tetrachlorodibenzo-*p*-dioxin, by activating AhR, is teratogenic by causing vitamin A deficiency [35]. Deletion of AhR resulted in RA accumulation in liver of mice and a lowered *Cyp2c39* transcript was ascribed to this defect [12]. Activated PXR induced expression of *CYP3A* and xenobiotic transporters, which accelerated RA metabolism [36]. Therefore, disturbed RA homeostasis and teratogenesis mediated by PO might be due to an interaction between these xenobiotic receptors being affected by PO (either directly or indirectly).

The safety concerns of frying food consumption generally focuses on cardiovascular diseases, cancers, and oxidative stress. This study highlights the potential impacts of OFO (or frying foods) on reproduction. In addition to disturbed RA metabolism, PO-mediated teratogenesis might be partially attributed to DNA mutation. As suggested by Indart et al. [37], aldehydes formed due to lipid oxidation are mutagenic and genotoxic, as they can covalently modify DNA to cause chromosomal aberrations. Although 25% PO in used oil is organoleptically unacceptable and should be discarded, a survey across EU show PO in frying oils from fast foods and restaurants ranging from 3%–60% [38]. Particular attention should be paid for discontinuous or intermittent heating, since it accelerates the deterioration of frying oils [39].

## 4. Materials and Methods

### 4.1. Preparation of PO

The OFO was prepared under a realistic experimental condition by frying dough sheets in soybean oil (President, Tainan, Taiwan) at 205 ± 5 °C for four 6-h intervals, as described [40]. Separation of PO from this OFO followed the Standard IUPAC Method 2.507 [41], as described [4]. The yield for the PO was 50% of the OFO. To evaluate the oil quality, acid value and conjugated diene concentrations of fresh soybean oil (FO) and PO were analyzed according to American Oil Chemists' Society methods [42].

### 4.2. Animals and Diets

Homozygous PPARα KO mice, on a pure C57BL/6J genetic background, and their wild-type (WT) control mice were purchased from Jackson Lab (Bar Harbor, ME, USA). Female and male WT or KO mice (8 weeks of age) were mated (within their genetic line) by overnight housing (ratio of 1 male to 3 females). Conception was confirmed by presence of a vaginal plug (the following morning) and was designated day1. Pregnant WT or KO females were fed a fresh soybean oil (FO) (group FO(WT) and FO(KO)) or PO (group PO(WT) and PO(KO)) diet, from day1 to day18. There were 5 dams in each group. Composition of the test diets followed AIN93M [43], with dietary fat increased from 4% to 10% (corn starch reduced from 62% to 56%) for FO or PO. All mice were kept in a room maintained at 23 ± 2 °C, with a controlled 12-h light:dark cycle with ad libitum access to food and drinking water. Body weight and food intake were recorded weekly. Pregnant mothers were killed on day18 (carbon dioxide asphyxiation). Aliquots of liver from dams and fetuses were recovered and stored at −80 °C for RNA extraction. All protocols for animal care and handling were approved by the Institutional Animal Care and Use Committee of China Medical University (103-69-N).

### 4.3. Embryonic Toxicity and Morphogenesis

During dissection, numbers of corpora lutea, implantations, and live/dead fetuses per litter were recorded. Fetuses were carefully removed from the uterus, weighed, and gross external morphology was examined under a dissecting microscope. Maternal weight gain, pre- and post-implantation loss/litter, were calculated as follows:

Maternal weight gain = Body weight at pregnancy day18 − Body weight at day0 − Uterus weight (including weight of fetuses and placentae) at day18.

Pre-implantation loss/litter = Number of corpora lutea/litter − Number of implantations/litter

Post-implantation loss (resorptions)/litter = Number of implantations/litter − Number of fetuses/litter.

### 4.4. Quantification of Retinol and Retinoic Acid

Due to the number of fetuses required for RA analysis, another batch of mice was treated as above (*n* = 5 for each group). The teratogenesis was reproduced by PO diet regardless of genotype [44] (data not shown). Extraction of retinoid from whole fetuses and maternal liver samples was done as described [45], with minor modifications. Four fetuses of the same group were pooled as one sample. One fetal sample or 0.5 g maternal liver was homogenized with 2 mL of phosphate buffer saline and methanol (2:1 *v*/*v*) mixture. Following homogenization, 250 μL of 0.5 N KOH in ethanol and 750 μL of ethanol was added, followed by 4 mL of hexane. After vortexing and centrifuging (15 min at 800× *g*) at room temperature, the hexane layer was collected. For the lower phase, 500 μL of 6N HCl and 4 mL of hexane were added. After vortexing and centrifuging, the hexane layer was collected and combined with the first hexane layer collected. The extract was dried under nitrogen, and re-suspended in 100 μL of methanol prior to injection into the HPLC.

The HPLC consisted of a Hitachi L-7100 Pump (Hitachi, Tokyo, Japan), a Hitachi L-7200 autosampler, a L-7100 UV detector, a FL detector L-7480, and a Vydac 201TP54 C18 reverse phase column (4.6 × 25 cm; Vydac, Hesperia, CA, USA). A gradient elution system was used for analysis of retinol and RA, as reported [46]. Retinol was determined by fluorescence with absorbance and emission (at 525 and 520 nm, respectively). RA was determined by UV with absorbance at 250 nm.

### 4.5. RNA Isolation and mRNA Detection

Liver (0.1 g from dam or 0.05 g from fetus) was homogenized in TRIZOL reagent (Invitrogen, Carlsbad, CA, USA) and total RNA was obtained according to the manufacturer’s instructions. Total RNA (1 μg) was reverse-transcribed into first-strand cDNA using 200 units of MMLV-RT (Promega) in a total volume of 20 μL. For real-time PCR, a SYBR system with self-designed primers (Table S1) and 12.5 ng cDNA was used. Amplification using 40 cycles of two steps (95 °C for 15 s and 60 °C for 1 min) was performed on an ABI Prism 7900HT sequence detection system (Foster City, CA, USA).

### 4.6. Statistical Analyses

Data were expressed as mean ± SEM. Fetal data was expressed on a litter basis, i.e., the abnormality frequency, retinoid concentration or gene expression level was calculated per litter and means ± SEM was obtained from 5 L per group. To determine the significance of the effects of diet (FO vs. PO) or genotype (WT vs. KO) and their interaction, data for the FO(WT), PO(WT), FO(KO), and PO(KO) groups were analyzed by two-way ANOVA. When there was a significant interaction between diet and genotype (*p*-interaction < 0.05), differences among the four groups were detected with one-way ANOVA and Duncan’s multiple range test. For incidence of litters with resorptions ≥3 and full-litter resorptions, the significance of differences between groups was analyzed using Chi-square. If variances were not homogeneous, data were log-transformed prior to statistical analysis. The General Linear Model of the SAS package (SAS institute, Cary, NC, USA) was used for all statistical analyses, and differences were considered significant at *p* < 0.05.

## 5. Conclusions

A deficiency of PPARα, by itself, increased RA concentration in the liver of pregnant mothers, but did not cause teratogenesis and fetal RA excess as long as they were fed a normal diet. PO-induced teratogenesis and disturbed RA homeostasis in fetuses were independent of PPARα activation. Furthermore, PO perturbed RA metabolism might have been due to crosstalk between several xenobiotic receptors.

## Figures and Tables

**Table 1:** Quality index of fresh soybean oil (FO) and polar compounds (PO) from oxidized frying oil.

Quality Index	FO	PO
Acid value, mg KOH/g	0.056 ± 0.002	2.9 ± 0.1
Conjugated diene, OD_233_/g	392 ± 4.23	4933 ± 35.52

**Table 2:** Embryotoxicity and reproductive characteristics of the wild (WT) and peroxisome proliferator-activated receptor α (PPARα) knock out (KO) females receiving FO or PO diets during pregnancy ^1,2^.

Variables	FO(WT)	PO(WT)	FO(KO)	PO(KO)	*p* Values (Two-Way ANOVA)		
					D	G	D × G
No. of dams	5	5	5	5			
Maternal weight gain, g	4.71 ± 0.52	2.97 ± 0.52	4.94 ± 0.86	1.98 ± 0.75	<0.005	NS	NS
Relative liver weight, %	4.58 ± 0.15 ^c^	7.35 ± 0.12 ^a^	5.11 ± 0.23 ^bc^	5.90 ± 0.28 ^b^	<0.0001	NS	<0.005
No. of corpora lutea/litter	7.00 ± 0.32	8.60 ± 0.68	9.40 ± 0.51	9.20 ± 0.73	NS	NS	NS
No. of implantations/litter	7.00 ± 0.51	8.60 ± 0.68	9.40 ± 0.51	9.20 ± 0.73	NS	NS	NS
No. of fetuses/litter	5.40 ± 0.68	6.60 ± 0.75	8.60 ± 0.75	6.80 ± 1.69	NS	NS	NS
No. of live fetuses/litter	5.40 ± 0.68	6.40 ± 0.75	8.40 ± 0.93	6.60 ± 1.57	NS	NS	NS
No. of dead fetuses/litter	0.00 ± 0.00	0.20 ± 0.20	0.20 ± 0.20	0.20 ± 0.20	NS	NS	NS
Pre-implantation loss/litter	0.00 ± 0.00	0.00 ± 0.00	0.00 ± 0.00	0.00 ± 0.00	NS	NS	NS
Post-implantation loss/litter	1.00 ± 0.32	2.00 ± 0.45	0.80 ± 0.58	2.40 ± 1.21	NS	NS	NS
Litters with resorptions ≥3, %	0 (0/5)	40 (2/5)	20 (1/5)	20 (1/5)			
Full-litter resorptions, %	0 (0/5)	0 (0/5)	0 (0/5)	0 (0/5)			

^1^ Values are means ± SEM. Two-way ANOVA was conducted and results are shown in table (D, diet; G, genotype; D × G, interaction; NS, not significant). When there was a significant interaction between D and G, the significance of differences among groups was further analyzed by one-way ANOVA and Duncan’s multiple range test; ^a–c^ Values without a common superscript letter differed (*p* < 0.05); ^2^ For the last two variables, the difference between groups was analyzed by χ^2^. There was no significant difference between FO and PO groups within the same genotype, either in WD or KO mice within the same diet.

**Table 3:** Body and tissue weight, mortality, and externally visible congenital anomalies of fetuses from the wild (WT) and PPARα knock out (KO) females receiving FO or PO diets during pregnancy ^1^.

Variables	FO(WT)	PO(WT)	FO(KO)	PO(KO)	*p* Values (Two-Way ANOVA)		
					D	G	D × G
No. of litter ^2^	5 (27)	5 (33)	5 (43)	5 (34)			
Body weight, g	0.81 ± 0.03	0.88 ± 0.05	0.80 ± 0.36	0.76 ± 0.07	NS	NS	NS
Placenta, g	0.07 ± 0.00	0.09 ± 0.00	0.09 ± 0.04	0.10 ± 0.01	<0.005	NS	NS
Liver, g	0.04 ± 0.00	0.04 ± 0.00	0.04 ± 0.02	0.04 ± 0.00	NS	NS	NS
Mortality rate, %	0 ± 0	3.4 ± 3.3	0 ± 0	2.8 ± 2.7	NS	NS	NS
**Incidence of externally visible abnormalities, %**							
Eye defect	0 ± 0	3.4 ± 3.3	0 ± 0	2.8 ± 2.7	NS	NS	NS
Edema	0 ± 0	3.4 ± 3.3	0 ± 0	3.4 ± 3.3	NS	NS	NS
Brain defect	0 ± 0	2.8 ± 2.7	0 ± 0	2.8 ± 2.7	NS	NS	NS
Haematoma	0 ± 0	8.8 ± 3.6	0 ± 0	20 ± 12	<0.05	NS	NS
Shrivelling	0 ± 0	12.4 ± 5.3	0 ± 0	11.2 ± 4.8	<0.05	NS	NS
Spina bifida	0 ± 0	3.4 ± 3.3	0 ± 0	6.2 ± 3.7	NS	NS	NS
Limb defect	0 ± 0	6.8 ± 4.0	0 ± 0	2.8 ± 2.7	NS	NS	NS
Total ^3^	0 ± 0	30.2 ± 8.4	0 ± 0	32.5 ± 9.7	<0.05	NS	NS

^1^ Values are means ± SEM. Two-way ANOVA was conducted and results are shown in table (D, diet; G, genotype; D × G, interaction; NS, not significant); ^2^ Total fetus number in parenthesis; ^3^ One fetus may have more than one abnormality.

## Supplementary Materials

Supplementary materials can be found at www.mdpi.com/1422-0067/18/3/510/s1.

## Abbreviations

AhR	Aryl hydrocarbon receptor
CAR	Constitutive androstane receptor
DHRS	Dehydrogenase/reductase SDR family
FO	Fresh oil
KO	Knock out
OFO	Oxidized frying oil
PO	Polar compounds
PPs	Peroxisome proliferators
PXR	Pregnane-X receptor
RA	Retinoic acid
RALDH	Retinal dehydrogenase
RAR	Retinoic acid receptor
RARE	Retinoic acid response element
RDH	Retinol dehydrogenase
RXR	Retinoid X receptor
WT	Wild type
