# Supplementary material for: Peroxisome Proliferator-Activated Receptor α Activation Is Not the Main Contributor to Teratogenesis Elicited by Polar Compounds from Oxidized Frying Oil
Source: Int J Mol Sci. 2017 Feb 27;18(3):510. doi: 10.3390/ijms18030510 (PMC5372526; doi:10.3390/ijms18030510)
Supplement: Supplementary file 1 [file ijms-18-00510-s001.pdf]

Supplementary Materials: Peroxisome Proliferator-Activated Receptor α Activation Is Not the Main Contributor to Teratogenesis Elicited by Polar Compounds from Oxidized Frying Oil

Yu-Shun Lin, Ting-Yi Lin, Jia-Jiuan Wu, Hsien-Tsung Yao, Sunny Li-Yun Chang and Pei-Min Chao

Table S1. Gene names and sequences of PCR primers.

Gene	Encoding protein	Accession Number	Primer
Acox	Acyl-CoA oxidase	NM_015729	F:CCAAGATTCAAGACAGAGCC<br>R:TCCCCTCAAGAAAATCCCC
Ahr	Aryl-hydrocarbon receptor	NM_013464.4	F:CGTCCCTGCATCCCACTACTT<br>R:GGACATGGCCCCAGCATAG
Car	Constitutive androstane receptor	NM_001243062.1	F:CCCTCTTCTCCCCTGGTTTC<br>R:AGCAGACAGTTCCTCCAAGC
Cyp1a1	Cytochrome P450, family 1, subfamily a, polypeptide 1, transcript variant 1	NM_009992.4	F:TCTCGTGGAGCCTCATGTACCT<br>R:TGCCGATCTCTGCCAATCA
Cyp2b10	Cytochrome P450, family 2, subfamily b, polypeptide 10	NM_009999.3	F:CACCACGCTCCGCTATGGCT<br>R:TTGGTAGCCGGTGTGAGCCG
Cyp2c39	Cytochrome P450, family 2, subfamily c, polypeptide 39	NM_010003.2	F:AGAGATTCATCAACCTTGTCCCTAA<br>R:GATGTCAGTGACGTTACTACTGTTGTTC

Table S1. *Cont.*

Gene	Encoding protein	Accession Number	Primer
Cyp3a11	Cytochrome P450, family 3, subfamily a, polypeptide 11	NM_007818.3	F:GGTCAAACGCCTCTCCTTGCTGT<br>R:CTGGGCCAAAATCCCGCCGGT
Cyp4a10	Cytochrome P450, family 4, subfamily a, polypeptide 10	NM_010011.3	F:TGAGGGAGAGCTGGAAAAGA<br>R:CTGTTGGTGATCAGGGTGTG
Cyp26a1	Cytochrome P450, family 26, subfamily a, polypeptide 1	NM_001105201.1	F:GAGCTGAAGGAGTTGGCTGTA<br>R:GATTTTGGTGATGGCTGCTGG
Cyp26b1	Cytochrome P450, family 26, subfamily b, polypeptide 1, transcript variant 1	NM_175475.3	F:AGCTAGTGAGCACCGAGTGG<br>R:GGGCAGGTAGCTCTCAAGTG
Cyp26c1	Cytochrome P450, family 26, subfamily c, polypeptide 1	NM_001105201.1	F:GAGCTGAAGGAGTTGGCTGTA<br>R:GATTTTGGTGATGGCTGCTGG
Pxr	Pregnane X receptor	NM_010936.3	F:TAAGCTGAGATCTCCATGTGC<br>R:TACATCTGTGTGTCCTAGACTGT
Raldh1	Retinaldehyde dehydrogenase 1	NM_013467.3	F:TGCGCATTGCCAAAGAGGAGATATT<br>R:CATCTTGAATCCACCGAAGGGGCA
Raldh2	Retinaldehyde dehydrogenase 2	NM_009022	F:GAGAGAAATGGGTGAGTTTGGC<br>R: GACCACGGGGTATGACGGAG
Raldh3	Retinaldehyde dehydrogenase 3	NM_053080	F:TTCAAAAACCTGGAGGAGGTGA<br>R:ATGCATTGTAGCAGTTGATCCAGA
Rdh10	Retinol Dehydrogenase 10	NM_133832.3	F:GCCACGCACACTTCTGGACCAC<br>R:GGAGCTCGCGACCGTCACAAT
Dhrs4	Dehydrogenase/reductase SDR family member 4	NM_001037938.2	TCAGCAGTGTGTTGTGGGAGGAGA<br>CGGTCTCTCCATTGATGTAACTGGC

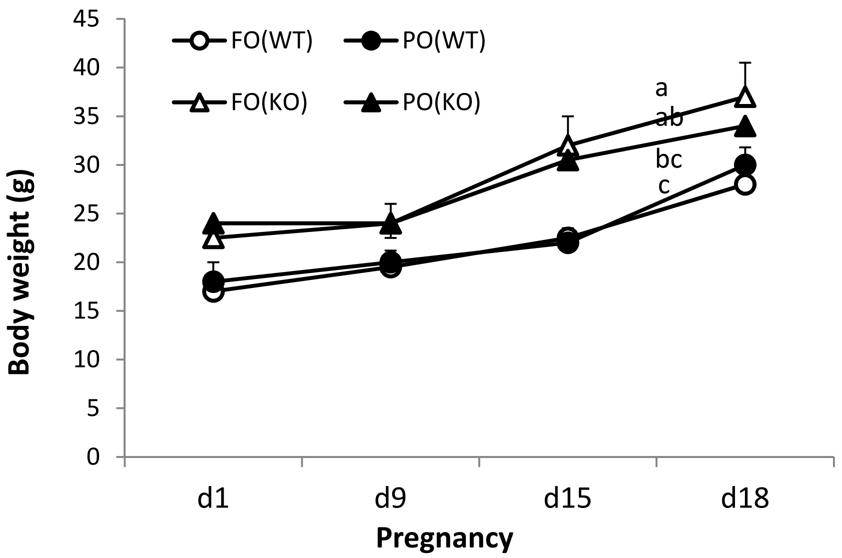

P for Two-Way ANOVA	Day1	Day9	Day15	Day18
D	NS	NS	NS	NS
G	<0.001	<0.001	<0.0001	<0.05
D × G	NS	NS	NS	<0.05

Figure S1. Body weight of the wild (WT) and PPARα knock out (KO) females receiving FO or PO diets during pregnancy. Data are mean ± SEM, n = 5. Results of two-way ANOVA are shown in table (D, diet; G, genotype; D × G, interaction; NS, not significant).

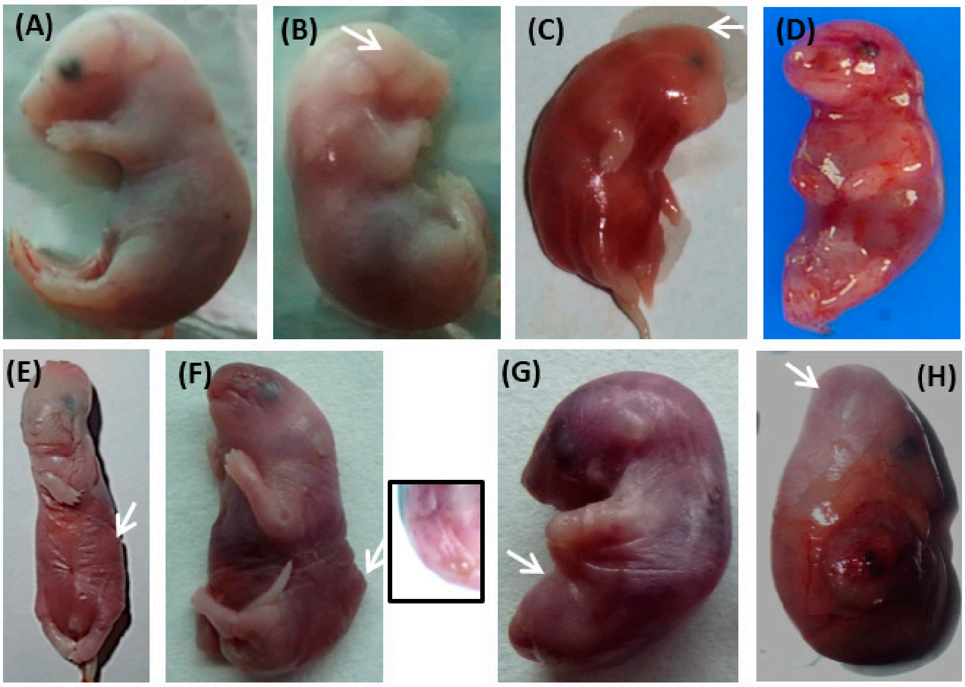

Figure S2. Abnormalities of external morphology of embryos from dams at pregnancy day18. (**A**) Normal; (**B**) Eye defect includes unilateral or bilateral anophthalmia; (**C**) Edema and brain defect; (**D**) Haematoma; (**E**) Surface shriveling; (**F**) Spina bifida; (**G**) Limb defects include missing of hind limbs; (**H**) Brain defect (or cranial deformity) includes anencephaly or microcephaly. Local abnormalities are indicated by arrows. The meningocele is seen for spina bifida as amplified in box.
